# Feline leishmaniosis in the Mediterranean Basin: a multicenter study

**DOI:** 10.1186/s13071-024-06419-x

**Published:** 2024-08-19

**Authors:** Mariaelisa Carbonara, Roberta Iatta, Guadalupe Miró, Ana Montoya, Giovanni Benelli, Jairo Alfonso Mendoza-Roldan, Elias Papadopoulos, Clara Lima, Emilie Bouhsira, Yaarit Nachum-Biala, Nicola Decaro, Bettina Schunack, Gad Baneth, Domenico Otranto

**Affiliations:** 1 Department of Veterinary Medicine, University of Bari, Bari, Italy; 2 Interdisciplinary Department of Medicine, University of Bari, Bari, Italy; 3 Animal Health Department, Veterinary Faculty, Universidad Complutense de Madrid, Madrid, Spain; 4 Department of Agriculture, Food and Environment, University of Pisa, Pisa, Italy; 5 Department of Infectious and Parasitic Diseases and Pathology, School of Veterinary Medicine, Aristotle University of Thessaloniki, Thessaloniki, Greece; 6 Department of Biological Sciences, Microbiology Laboratory, Faculty of Pharmacy, University of Porto, Porto, Portugal; 7 InTheres, Université de Toulouse, INRAE, ENVT, Toulouse, France; 8 School of Veterinary Medicine, Hebrew University, Rehovot, Israel; 9 Elanco Animal Health, Monheim, Germany; 10 Department of Veterinary Clinical Sciences, City University of Hong Kong, Hong Kong SAR, China

**Keywords:** *Leishmania infantum*, Domestic cats, Risk factors, Clinical signs, Clinicopathological abnormalities, Mediterranean Basin countries

## Abstract

**Background:**

Cats are now recognized as competent hosts for *Leishmania infantum* and a blood source for sand fly vectors. Although canine leishmaniosis (CanL) is endemic in Mediterranean Basin countries, large-scale epidemiological studies are lacking for feline leishmaniosis (FeL). This study aimed to assess the prevalence of *L. infantum* infections, associated risk factors, clinical signs, and clinicopathological abnormalities in domestic cat populations from six Mediterranean Basin countries.

**Methods:**

From 2019 to 2022, blood and serum samples of cats (*n* = 2067) living in Italy (*n* = 300), Greece (*n* = 297), Portugal (*n* = 295), France (*n* = 231), Israel (*n* = 313), and Spain (*n* = 631) were collected along with animal data (i.e., age, sex, breed, housing conditions, and geographical origin), clinical signs, and laboratory blood test parameters. Cats were grouped according to their age as kittens (up to 1 year), young (older than 1 and younger than 7 years), mature (between 7 and 10 years), and senior (older than 10 years). Serum samples were tested for *L. infantum* by immunofluorescence antibody test (IFAT) and enzyme-linked immunosorbent assay (ELISA), and blood samples of seropositive cats were tested for *L. infantum* kinetoplast deoxyribonucleic acid (kDNA). Viral infection by feline immunodeficiency virus (FIV) and feline leukemia virus (FeLV) was molecularly addressed in all cats enrolled. Statistical analysis was performed to evaluate the association between the risk of *L. infantum* infection and independent variables, and among co-infection of *L. infantum* with FIV and/or FeLV, clinical signs, and clinicopathological abnormalities.

**Results:**

Overall, 17.3% (358/2067) of cats scored positive for *L. infantum* by serological tests. Specifically, 24.7% were from Portugal, 23.2% from Greece, 16.6% from Israel, 15% from Spain, 13.3% from France, and 12.6% from Italy. *Leishmania infantum* DNA was detected in 15 seropositive animals. Housing condition and FIV infection proved to be risk factors for FeL. *Leishmania* seropositivity was significantly associated with weight loss, lymphadenomegaly, gingivostomatitis, and oral ulcers, as well as with reduced albumin and albumin/globulin ratio, increased total globulins and total proteins, leukocytosis, and thrombocytosis.

**Conclusions:**

This study provides, for the first time, a large-scale epidemiological survey on FeL and its clinical presentation, revealing that *L*. *infantum* circulates among domestic cats, especially shelter/free-roaming and FIV-infected animals, living in CanL endemic countries of the Mediterranean Basin.

**Graphical Abstract:**

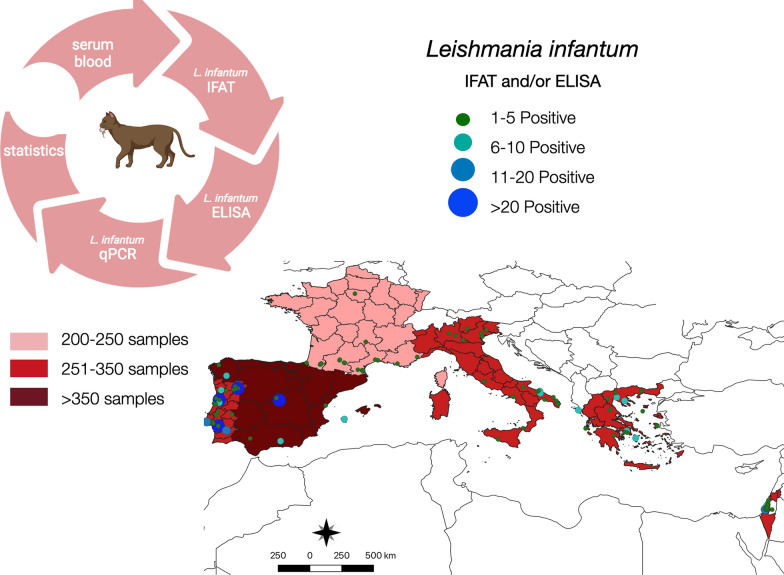

## Background

*Leishmania infantum* (Kinetoplastida: Trypanosomatidae) is one of the most important zoonotic vector-borne pathogens transmitted by sand flies (Diptera: Psychodidae) [1] and is widespread in countries of the Mediterranean Basin, Middle East, western Asia, and Brazil [2, 3]. Dogs, the main reservoirs of this protozoan parasite, are fundamental for the circulation of the infection in endemic areas, with a significant proportion of them not showing any clinical sign or presenting few and often non-specific symptoms [4–6]. Cats have long been considered less susceptible hosts for *L. infantum*; however, they are now recognized as competent hosts for this parasite and a blood source for its sand fly vectors [7–9]. Accordingly, in recent decades, many cases of feline leishmaniosis (FeL) have been described in regions endemic for canine leishmaniosis (CanL) (e.g., Italy, Spain, Greece, Portugal, Cyprus, Israel, Brazil), with prevalence ranging from 0.7% to 70%, depending on animal lifestyle, number of animals tested, and diagnostic technique employed [10, 11]. In addition, in Brazil, there are reports on *L. infantum*-infected cats in 12 out of 27 states [9], highlighting a potential reservoir role of these felids [12, 13]. Under specific ecological habitats (e.g., animal shelters) and in the presence of CanL, cats can be significantly more exposed to *L. infantum* infection (i.e., 75%) than dogs (i.e., 37%). Notwithstanding, they present lower parasitemia [6]. Overall, studies performed in endemic regions, point out a lower prevalence of FeL when compared with CanL from the same area [14]. For example, in the Aeolian Islands (Sicily, Italy), a Mediterranean region highly endemic for CanL, a 25.8% seroprevalence of FeL was reported, being about half of the prevalence recorded in dogs (i.e., 41.8%) [15].

Although scientific information about FeL has increased in the past few years, many clinical and diagnostic challenges remain unsolved, hindered by a broad spectrum of clinical signs and laboratory abnormalities described in the literature [16–18]. Among FeL diseased animals, unspecific clinical observations (i.e., lymphadenomegaly, weight loss, and pale mucous membranes) are often associated with cutaneous (i.e., exfoliative and ulcerative dermatitis, nodules, onychogryphosis, nasal/footpad hyperkeratosis) and ocular lesions (i.e., keratoconjunctivitis, uveitis), alongside hyperglobulinemia and mild/moderate non-regenerative anemia [11, 18]. In addition, infection with the feline immunodeficiency virus (FIV) and/or feline leukemia virus (FeLV) may predispose cats to becoming co-infected with *Leishmania* and developing FeL [8, 19]. Nonetheless, the subclinical presentation of FeL may challenge its diagnosis, which usually requires a combined approach using direct and indirect *Leishmania*-specific laboratory tests [20, 21]. Qualitative/quantitative immunofluorescence antibody test (IFAT) and enzyme-linked immunosorbent assay (ELISA) are the most common serological techniques used for both diagnosis and epidemiological studies [22–24]. Similarly, molecular investigations (e.g., real-time polymerase chain reaction [qPCR], conventional polymerase chain reaction [cPCR]) are widely employed in research and clinical practice [12, 25].

Given the endemicity of CanL in the Mediterranean Basin, the increasing number of FeL cases recorded, and the absence of epidemiological studies carried out on a large scale, this study aimed to assess the prevalence of *L. infantum* infection, associated risk factors, and clinical-hematological abnormalities in domestic feline populations from six countries of the Mediterranean Basin.

## Methods

### Study locations

From 2019 to 2022, domestic cats (*n* = 2067) were sampled by six veterinary academic institutions in southern European countries, specifically Italy (*n* = 300), Greece (*n* = 297), Portugal (*n* = 295), France (*n* = 231), Israel (*n* = 313), and Spain (*n* = 631). Approximately 300 cats were sampled per country, using the same enrollment criteria. One exception was Spain, where the number of enrolled animals was double that in the other countries, in proportion to country’s geographical dimensions, and given the fact that it presents one of the areas with the highest CanL endemicity in the studied region [26, 27]. Serum and blood samples were sent to the Parasitology Unit of the Department of Veterinary Medicine, University of Bari (Italy), for IFAT and molecular analysis. Individual aliquots of each serum sample (approximately 20 μl) were sent to the School of Veterinary Medicine, Hebrew University, Israel, for in-house ELISA testing. Whenever serum volume was insufficient, only IFAT was performed.

### Inclusion criteria

All the animals included in the study had a history of outdoor access and had not been treated with ectoparasiticides or repellents for at least 6 months prior to sampling. At enrollment, signalment data (i.e., age, sex, breed), housing conditions, and the geographical area (zip code, city/town) for each cat were recorded in individual files. In addition, health status and laboratory parameters, including complete blood cell count (CBC) and serological biochemical parameters (i.e., creatinine, urea, alanine aminotransferase [ALT], albumin, total proteins, total globulins, and albumin/globulin [A/G] ratio, were recorded, when available, in individual clinical cards. Clinical data were summarized as follows: general signs (i.e., fever, pale mucous membranes, hepatomegaly, weight loss, jaundice, asthenia, anorexia, lymphadenomegaly), skin signs (i.e., ulcers/crusts/scales, dandruff, nodules/hemorrhagic cysts, alopecia, squamous cell carcinoma), ocular signs (i.e., blepharitis, keratitis, conjunctivitis, uveitis), oral signs (i.e., gingivostomatitis, mouth ulcers), gastrointestinal signs (i.e., vomiting, diarrhea), respiratory signs (i.e., dyspnea, nasal discharge, ocular discharge), and renal sign (i.e., chronic kidney disease [CKD]). Specifically, at least 10% of the cats sampled from each geographical area had clinicopathological information. Cats enrolled were grouped according to their age as kitten (up to 1 year), young (older than 1 and younger than 7 years), mature (between 7 and 10 years), and senior (older than 10 years) [28].

### Serological testing

Serum samples were tested for antibodies against anti-*L. infantum* by means of IFAT and ELISA. The IFAT was carried out at the Parasitology Unit, Department of Veterinary Medicine of Bari University (Italy), following a previously described protocol [24]. Positive and negative controls included serum samples from a cat infected with *L. infantum*, previously diagnosed using IFAT and molecular assays (i.e., qPCR), and serum samples from healthy cats previously tested negative by the methods described, respectively. A sample was scored positive whenever it produced a clear promastigote fluorescence at a cut-off dilution of 1:80, as currently recommended by the LeishVet guidelines [20]. Positive serum samples were titrated by serial dilutions until negative results were obtained. All IFAT tests were read in a double-masked manner by two different operators.

Serum samples were tested by ELISA at the School of Veterinary Medicine, Hebrew University, Rehovot, Israel, using crude leishmanial antigen, as described previously [6]. Each plate was read when the absorbance (lambda = 405 nm) of the positive cat reference serum reached a value between 1.1 and 1.2. A titration of positive and negative reference cat sera was included on each plate to monitor inter-assay variation. The serological cut-off of optical density (OD) = 0.6 was calculated based on three standard deviations above the mean OD value of readings from 13 control serum samples from seronegative and PCR-negative cats living in a non-endemic region.

### Molecular testing

Deoxyribonucleic acid (DNA) was extracted from 200 μl of whole blood using a commercial kit (QIAamp DNA Blood & Tissue Kit, Qiagen, Hilden, Germany) according to the manufacturer’s instructions and analyzed for the detection of FIV and FeLV proviral DNA by PCR using primers and protocol described previously [29, 30]. Whole blood DNA samples of seropositive (by IFAT and/or ELISA) cats were further tested by qPCR for the detection of a fragment (120 base pairs) of the *L. infantum* kinetoplast deoxyribonucleic acid (kDNA) minicircle, using primers, probes, and protocols described elsewhere [31]. For all qPCR runs, positive (i.e., DNA extracted from a blood sample of a cat molecularly and serologically positive for *L. infantum*) and negative controls (DNA extracted from blood samples of negative healthy cats) were included. Samples were scored as positive for *L. infantum* kDNA when a threshold cycle of less than 37 was recorded.

### Mapping and statistical analysis

The cumulative prevalence of *Leishmania* infection was calculated by summing the seropositivity by IFAT and ELISA. The 95% confidence interval (CI) values were calculated for each prevalence recorded using Epitools - Epidemiological Calculators software [32]. Agreement between IFAT and ELISA test results was evaluated by Cohen’s kappa test. The location of *L. infantum*-positive cats was georeferenced using a geographical information system (GIS) program (QGIS software, Buenos Aires version).

Feline categorical data were summarized as count and percentage. Comparisons between independent groups were performed by the chi-squared test. Differences in cumulative serological positivity in cats of different sexes, ages, housing conditions, and countries, as well as of individuals infected or not by FIV and FeLV, were analyzed using a generalized linear model with a binomial error structure (1 = serological positivity, 0 = negativity) and a fixed factor: *y* = *Xß* + *ε*, where *y* is the vector of the observation (i.e., the cat serological positivity for *L. infantum*), *X* is the incidence matrix, *ß* is the vector of fixed effects (i.e., housing condition, sex, age, country of origin, FIV or FeLV infection), and *ε* is the vector of the random residual effects. A probability level of *P* < 0.05 was used to assess the significance of differences among values.

Contingency analyses assessing the potential relationship between the cumulative serological positivity (yes/no) and selected clinical signs (i.e., fever, pale mucous membranes, hepatomegaly, weight loss, jaundice, asthenia, anorexia, lymphadenomegaly, ulcers/crusts/scales, dandruff, nodules/hemorrhagic cysts, alopecia, squamous cell carcinoma, blepharitis/keratitis/conjunctivitis/uveitis, gingivostomatitis, mouth ulcers, vomiting, diarrhea, dyspnea, nasal discharge, ocular discharge, and CKD), as well as between cumulative serological positivity and laboratory abnormalities in selected biochemical parameters (i.e., albumin, total globulins, ALT, creatinine, total proteins, urea, red blood cells [RBC], white blood cells [WBC], hematocrit [Hct], hemoglobin [Hgb], platelet count [PLT], and A/G ratio) were conducted. All analyses were carried out using JMP 17 software (SAS Institute Inc., Cary, NC, USA).

## Results

Out of 2067 cats enrolled, the majority were young (60%) and common European breed (93.9%), with similar ratios of sex (49.2% female vs. 50.8% male) and housing condition (49.8% shelter/free-roaming vs. 50.2% owned) (Table 1). The percentages of shelter/free-roaming cats sampled per geographical area are detailed in Table 2.

**Table 1:** Comparison of serological and molecular prevalence of *Leishmania infantum* infections with animal data

Variables	Total no. of cats (2067) (%)	IFAT no. positive/no. of cats examined (%)	IFAT no. of positive (titers)	ELISA no. of positive/no. of cats examined (%)	qPCR no. of positive/no. of seropositive cats examined (%)	Total no. of positive cats (%)
Age						
Kittens	493 (23.8)	65/492 (13.2)	36 (80); 22 (160); 4 (320); 2 (640); 1 (1280)	42/453 (9.3)	1/70 (1.4)	74 (15)
Young	1242 (60)	200/1234 (16.2)	109 (80); 64 (160); 16 (320); 4 (640); 4 (1280); 3 (2560)	119/1160 (10.3)	10/202 (4.9)	228 (18.4)
Adults	169 (8.2)	28/170 (16.5)	13 (80); 11 (160); 3 (320); 1 (1280)	21/161 (13)	3/30 (10)	34 (20.1)
Seniors	163 (7.9)	19/163 (11.6)	11 (80); 7 (160); 1 (2560)	11/149 (7.4)	1/18 (5.5)	22 (13.5)
Gender						
Female	1018 (49.2)	154/1013 (15.2)	86 (80); 50 (160); 9 (320); 4 (640); 2 (1280); 3 (2560)	91/949 (9.6)	9/157 (5.7)	172 (16.9)
Male	1049 (50.7)	158/1040 (15.2)	83 (80); 54 (160); 14 (320); 2 (640); 4 (1280); 1 (2560)	102/975 (10.5)	6/163 (3.7)	186 (17.7)
Housing condition						
Shelter/free-roaming	1019 (49.3)	176/1013 (17.4)	91 (80); 59 (160); 17 (320); 4 (640); 3 (1280); 2 (2560)	117/979 (11.9)	10/182 (5.5)	206 (20.2)
Owned	1048 (50.7)	136/1046 (13)	78 (80); 45 (160); 6 (320); 2 (640); 3 (1280); 2 (2560)	76/945 (8)	5/138 (3.6)	152 (14.5)
Breed						
European	1969 (95.2)	305/1961(15.5)	165 (80); 101 (160); 23 (320); 6 (640); 6 (1280); 4 (2560)	189/1832 (10.3)	15/311 (4.8)	348 (17.7)
Non-European	98 (4.7)	7/97 (7.2)	4 (80); 3 (160)	4/92 (4.3)	0/9	10 (10.2)
Country						
Spain	631 (30.5)	72/626 (11.5)	34 (80); 25 (160); 6 (320); 3 (640); 3 (1280); 1 (2560)	66/614 (10.7)	8/86 (9.3)	95 (15.5)
Italy	300 (14.5)	33/300 (11)	22 (80); 7 (160); 1 (640); 1 (1280); 2 (2560)	19/276 (6.8)	3/38 (7.9)	38 (12.6)
Greece	297 (14.4)	65/297 (21.8)	33 (80); 29 (160); 2 (320); 1 (1280)	39/244 (16)	0/68	69 (23.2)
Israel	313 (15.1)	49/313 (15.6)	31 (80); 14 (160); 4 (320)	19/277 (6.8)	0/52	52 (16.6)
Portugal	295 (14.3)	69/295 (23.3)	31 (80); 24 (160); 11 (320); 1 (640); 1 (1280); 1 (2560)	38/295 (12.8)	4/64 (6.2)	73 (24.7)
France	231 (11.2)	24/228 (10.5)	18 (80); 5 (160); 1 (640)	12/218 (5.5)	0/24	31 (13.4)

**Table 2:** Serological (IFAT, ELISA) prevalence of *Leishmania infantum* infections in shelter/free-roaming and owned animals per geographical area

Country	No. of positive/no. of shelter/free-roaming cats (%)	No. of positive/no. of owned cats (%)
Spain	85/492 (17.3)	10/139 (7.2)
Italy	11/56 (19.6)	27/244 (11)
Greece	25/104 (24)	44/193 (22.8)
France	9/58 (15.5)	22/173 (12.7)
Israel	4/17 (23.5)	48/296 (16.2)
Portugal	72/292 (24.6)	1/3 (33.3)

**Fig. 1:**
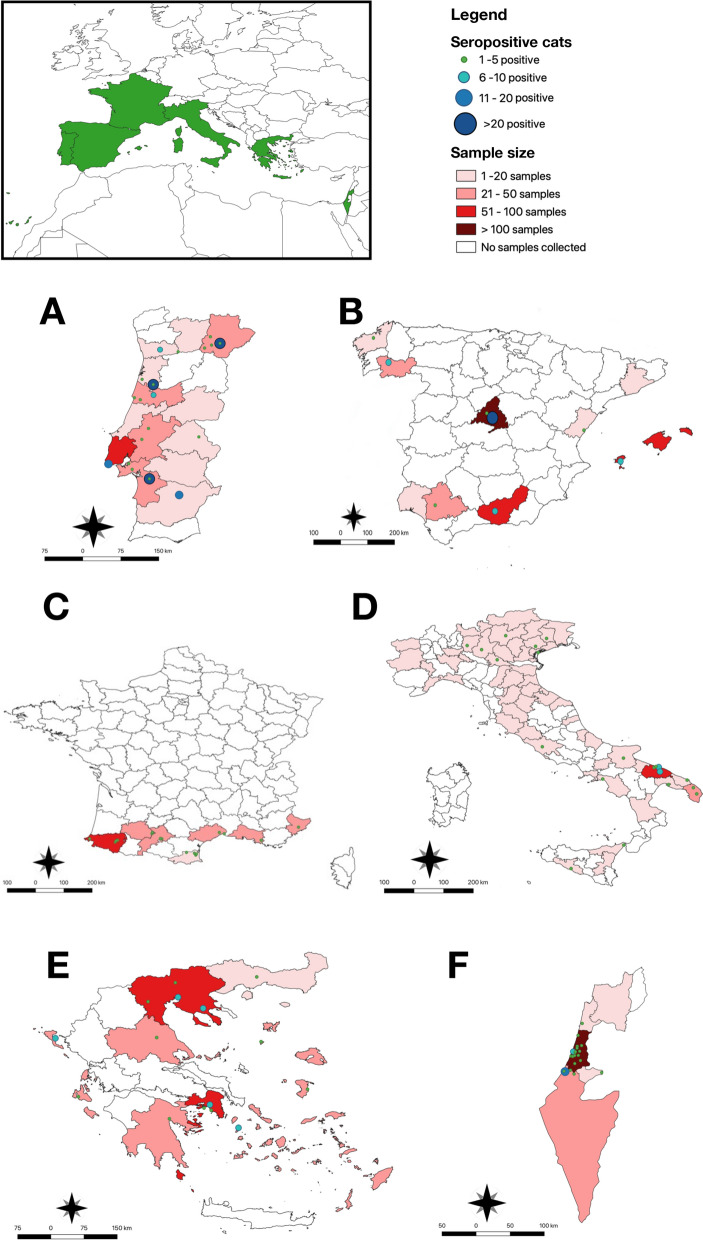
Map of study areas (i.e., **A** Portugal, **B** Spain, **C** France, **D** Italy, **E** Greece, **F** Israel) indicated by provinces, showing the sample size of cats enrolled, according to their *Leishmania infantum* seropositivity

Overall, 17.3% (358/2,067, 95% CI 15.7–19) of cats scored positive for *L*. *infantum* by serological tests. Specifically, 24.7% (73/295; 95% CI 20–29.9) were from Portugal, 23.2% (69/297; 95% CI: 18.7–28.3) from Greece, 16.6% (52/310; 95% CI 13.3–21.7) from Israel, 15% (95/631; 95% CI 12.5–18) from Spain, 13.3% (31/231; 95% CI 9.6–18.4) from France, and 12.6% (38/300; 95% CI 9.3–16.9) from Italy. The seroprevalence of *L*. *infantum* in shelter/free-roaming and owned animals per geographical area is depicted in Table 2. Of the 358 *L*. *infantum*-seropositive animals, 15.1% (312/2,059; 95% CI 13.6–16.7) tested positive by IFAT and 10% (193/1,924, 95% CI 8.7–11.4) by ELISA, with fair agreement between the two serological techniques, κ agreement = 0.246 (95% CI 19.7–29.5). A total of 7.4% (154/2,067; 95% CI 6.4–8.6) of animals were positive for both serological tests. Out of 358 cats seropositive by IFAT and/or ELISA, *L. infantum* DNA was detected in only 15 animals (4.6%; 95% CI 2.5–6.8). Of the IFAT-positive cats, 54.1% (169/312) had an antibody titer of 1:80, and 33.3% (104/312) of 1:160; in the remaining animals the titers varied from 1:320 to 1:5120 (Table 1). The seroprevalence of *L*. *infantum* in relation to animal data (i.e., age, gender, housing condition, breed, and geographical area) and clinical-hematological information is reported in Tables 1 and 3, respectively. The GIS analysis (Fig. 1) showed a scattered-coastal distribution of FeL.

**Table 3:** Health status, laboratory parameters, and retrovirus infections in the feline population studied

Health status data	Total no. of cats (%)	Tot no. positive cats (%)
Clinical signs	1569	281
Yes	417 (26.6)	75 (26.7)
No	1152 (73.4)	206 (73.3)
Systemic signs	260 (16.6)	62 (22)
Skin lesions	127 (8)	33 (11.7)
Ocular signs	73 (4.6)	11 (3.9)
Oral signs	88 (5.6)	26 (9.2)
Gastrointestinal signs	78 (5)	12 (4.2)
Respiratory signs	103 (6.6)	19 (6.8)
Urinary signs	34 (2.2)	6 (2.1)
Laboratory parameter data	Tot no. of cats (%)	Tot no. positive cats (%)
Clinicopathological abnormalities	793	151
Yes	763 (96.2)	148 (98)
No	30 (3.8)	3 (2)
Hematological parameters		
Hematocrit (28–43%)	730	141
High	111 (15.2)	23 (16.3)
Low	146 (20)	29 (20.6)
Normal	473 (64.8)	89 (63.1)
Leukocytes (5.5–12×1000/l)	732	141
High	317 (43.3)	77 (54.6)
Low	69 (9.4)	9 (6.4)
Normal	346 (47.3)	55 (39)
Platelets (130–400×100,000/ul)	722	141
High	140 (19.4)	34 (24.1)
Low	117 (16.2)	15 (10.6)
Normal	465 (64.3)	92 (65.2)
Biochemical parameters		
Total proteins (5.8–7.7 g/dl)	654	133
High	214 (32.7)	59 (44.4)
Low	25 (3.8)	2 (1.5)
Normal	415 (63.4)	72 (54.1)
Albumin (2.8–3.7 g/dl)	696	136
High	167 (24)	20 (14.7)
Low	164 (23.6)	46 (33.8)
Normal	369 (53)	70 (51.5)
Total globulins (2.9–4.3 g/dl)	656	131
High	248 (37.8)	70 (53.4)
Low	80 (12.2)	4 (3)
Normal	328 (50)	57 (43.5)
Albumin/globulin (0.6–1.3)	655	131
High	80 (12.2)	5 (3.8)
Low	150 (22.9)	44 (33.6)
Normal	425 (64.9)	82 (62.6)
Urea (29–60 mg/dl)	669	133
High	94 (14)	18 (13.5)
Low	159 (23.8)	24 (18)
Normal	416 (62.2)	91 (68.4)
Creatinine (0.93–1.7 mg/dl)	758	147
High	177 (23.3)	30 (20.4)
Low	168 (22.2)	25 (17)
Normal	413 (54.5)	92 (62.6)
Alanine transaminase (33–70 UI/l)	676	136
High	216 (32)	51 (37.5)
Low	86 (12.7)	14 (10.3)
Normal	374 (55.3)	71 (52.2)
Retrovirus infection data	Tot no. of cats (%)	Tot no. pos cats (%)
	2067	358
FIV	116 (5.6)	32 (8.9)
FeLV	48 (2.3)	4 (1.1)
FIV and FeLV	3 (0.1)	0

The risk of *L. infantum* infection in cats was significantly associated with housing condition (i.e., shelter/free-roaming animals, *χ*^2^ = 8.865, *df* = 1, *P* = 0.003), FIV infection (*χ*^2^ = 9.190, *df* = 1, *P* = 0.002), and country (*χ*^2^ = 31.009, *df* = 5, *P* < 0.0001) (Table 4). Concerning the country of origin, significant differences in cumulative serological positivity were detected between cats living in Greece and those in France (*χ*^2^ = 8.360, *df* = 1, *P* = 0.017), Italy (*χ*^2^ = 7.130, *df* = 1, *P* = 0.007), and Spain (*χ*^2^ = 8.360, *df* = 1, *P* < 0.0001); in addition, animals from Portugal had a higher risk of infection than those from France (*χ*^2^ = 5.733, *df* = 1, *P* = 0.003), Spain (*χ*^2^ = 13.777, *df* = 1, *P* < 0.0001), and Italy (*χ*^2^ = 4.784, *df* = 1, *P* = 0.029). Even if the generalized linear model showed no significance when testing the cat’s age as a risk factor (*χ*^2^ = 7.263, *df* = 3, *P* = 0.064), subsequent single contrasts outlined a significant difference between some age groups, i.e., 1–6 years vs. < 1 year (*χ*^2^ = 4.287, *df* = 1, *P* = 0.038), and 7–10 years vs. < 1 year (*χ*^2^ = 6.410, *df* = 1, *P* = 0.011).

**Table 4:** Risk factors associated with *Leishmania infantum* infection in cats

Risk factor	*df*	*X*^2^	*P*-value
Housing condition	1	8.865	0.003*
Sex	1	1.024	0.312
Age	3	7.263	0.064
Country	5	31.009	< 0.0001*
FeLV	1	3.605	0.058
FIV	1	9.190	0.002*
Breed	1	3.637	0.076

Out of 358 positive cats, health status data were available for 281 animals, of which 26.6% presented at least one clinical sign, with systemic features (i.e., 22%, 62/281) being the most common. Specifically, *L. infantum* seropositivity was significantly associated with weight loss (*χ*^2^ = 5.178, *df* = 1, *P* = 0.023), lymphadenomegaly (*χ*^*2*^ = 9.508, *df* = 1, *P* = 0.002), gingivostomatitis (*χ*^*2*^ = 5.701, *df* = 1, *P* = 0.017), and mouth ulcers (*χ*^*2*^ = 7.284, *df* = 1, *P* = 0.002). Complete blood cell count and serological biochemical parameters were available for 151 positive cats, of which 148 (i.e., 98%, 148/151) presented at least one clinicopathological alteration. Leukocytosis (i.e., 54.6%, 77/141) and increased total globulins and protein values (i.e., 53.5% and 44.4%, respectively) were the most frequent alterations recorded. Reduced albumin (*χ*^2^ = 12.915, *df* = 1, *P* = 0.002), increased total globulins (*χ*^2^ = 23.270, *df* = 1, *P* < 0.0001), increased total proteins (*χ*^2^ = 11.110, *df* = 1, *P* = 0.004), leukocytosis (*χ*^2^ = 7.132, *df* = 1, *P* = 0.028), thrombocytosis (*χ*^2^ = 10.207, *df* = 1, *P* = 0.006), and reduced A/G ratio (*χ*^2^ = 24.453, *df* = 1, *P* < 0.0001) were significantly associated with *L. infantum* positivity. For the above-mentioned significant parameters, the association between laboratory alteration scores and *L. infantum* seropositivity is detailed in Fig. 2.

**﻿Fig. 2:**
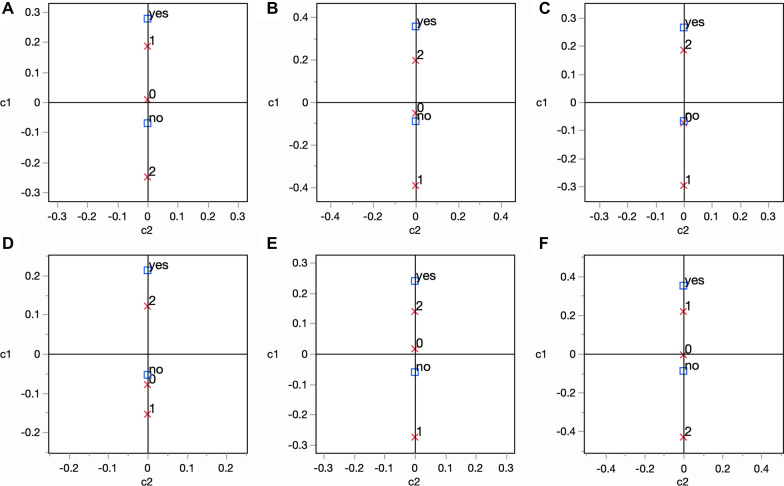
Correspondence analysis showing the association between clinicopathological abnormality scores [**A** albumin; **B** total globulins; **C** total proteins; **D** white blood cells, WBC; **E** platelet count, PLT; **F** albumin/globulin (A/G) ratio] and *L. infantum* seropositivity in cats. Each clinicopathological abnormality score for selected parameters is indicated by a red cross, whose distance from 0 and closeness to “yes” or “no” serological cumulative positivity indicate an association

## Discussion

The data presented indicate that *L*. *infantum* circulates within domestic feline populations living in countries of the Mediterranean Basin, where CanL is endemic. Indeed, this study collectively represents the first large-scale epidemiological survey on FeL conducted using the same procedures and diagnostic protocols. Hence, the overall seroprevalence of *L*. *infantum* recorded herein (17.3%) is not comparable with that derived from previous epidemiological studies conducted in individual countries, and often at a regional level [10, 11]. Nonetheless, it is known that sand fly vectors of *L. infantum* (i.e., *Phlebotomus ariasi, Phlebotomus neglectus, Phlebotomus perfiliewi, Phlebotomus perniciosus*) are present in the investigated areas [6, 33–37], and they may have cats as a blood source. Accordingly, a statistical positive association between *Leishmania* infection and antibody response to *P*. *perniciosus* saliva was described in cats [38], and cat blood was detected in *P*. *perniciosus* specimens from Spain [39–41] and Italy [42]. The *L. infantum* seroprevalence herein recorded in cats from each individual country is higher than in most of the previous studies, probably because it derives from the combination of IFAT and ELISA results and not just one serological test, as before [11]. Nonetheless, the seropositivity rates detected in Israel (16.6%), Spain (15%), and Italy (12.6%) are consistent with those retrieved in other studies from the same areas [43–49]. Likewise, the prevalence recorded in France (i.e., 13.3%) is similar to that reported in the only epidemiological investigation available from this country (i.e., 12%) [50], although the pathogen circulation was confirmed in many clinical case reports [51–53]. On the other hand, the rates of *Leishmania*-positive cats from Greece (i.e., 23.2%) and Portugal (i.e., 24.7%) are higher than those of the other investigated countries. At least for Portugal, the above picture may be related to the feline population sampled, which was mostly represented by shelter or free-roaming animals (i.e., 98.9%) (Table 2). Indeed, an outdoor lifestyle favors the animal’s exposure to sand fly bites, compared to individual housing [54, 55], as also suggested in a longitudinal study conducted in the Aeolian Islands [15].

The scattered-coastal distribution of FeL herein described may be related to the humid climate conditions of the investigated areas, which are suitable for sand flies to thrive [33]. This indicates the importance of regular use of repellents in cats living in seaside environments, to reduce the risk of sand fly bites [56]. In Israel, the distribution of FeL overlaps that of CanL in the central region of the country [57]. Overall, the seroprevalence of *L. infantum* in cats from each country is about half of that in dogs from the same areas (i.e., Italy: 12.6% in cats vs. up to 29.6% in dogs; Spain: 15% vs. up to 57%; Portugal: 24.7% vs. up to 56%; France: 13.3% vs. up to 29.6%; Israel: 16.6% vs. up to 36%; Greece: 23.2% vs. up to 50%) [6, 26, 36, 58–60]. This ratio was previously discussed in different epidemiological contexts [15, 48], alluding to the possibility of cats playing a less important role than dogs in the perpetuation of the *L. infantum* life cycle. However, these data should be interpreted cautiously, considering the difficulties in comparing animal populations, species-specific host–parasite interactions, and the accuracy of the available diagnostic tests. Indeed, one of the major challenges encountered in critically assessing *Leishmania* seroprevalence lies in the lack of consensus among techniques used in laboratories worldwide (i.e., IFAT, ELISA, western blot, direct agglutination test [DAT]) and cut-off values [14, 24, 61, 62]. Given the absence of standardized FeL screening tests, both IFAT and ELISA were used, having a fair agreement (κ agreement = 0.246), as previously suggested [62–64]. Accordingly, IFAT has a higher sensitivity than ELISA in the detection of subclinical/asymptomatic feline infections [62]. Therefore, the first technique is recommended for epidemiological purposes aiming to detect the exposure of cats to *Leishmania*, even if clinically healthy. Conversely, ELISA should be preferred in animals presenting signs suggestive of disease [62, 64]. As more than half of the IFAT-positive cats (i.e., 54%) had low antibody titers (1:80), these animals may have been exposed to *L. infantum* and/or may have generated a protective (Th1) immunity towards the parasite [65]. This is also supported by the high percentage of seropositive cats (i.e., 74%) with no clinical signs. Moreover, the detection of *L. infantum* DNA in blood samples of only a few individuals confirmed that blood is not a proper biological sample for *Leishmania* diagnosis, given the low parasitemia in the feline host [5, 6, 66]. Overall, future research should investigate the immune response of cats to *L. infantum* infection and validate the application of molecular tools with non-invasive feline samples (i.e., conjunctival swabs), as has been done for the diagnosis of CanL [67–69].

Shelter/free-roaming cats had a higher risk of infection than owned cats (*P* = 0.003), reflecting a higher exposure to phlebotomine sand flies, but also to several adverse environmental conditions (e.g., adverse weather conditions, poor nutritional state) that may impair their health status and favor *Leishmania* infection. In addition, FIV infection was found to be a risk factor for FeL (*P* = 0.002), as immunocompromised cats might be more prone to developing FeL clinical signs [8, 70–72]. A similar picture was found in human patients with HIV and co-infected with *Leishmania* [73]. Therefore, FIV infection should be considered within the diagnostic algorithm of *Leishmania* infection and for evaluating the prognosis of the disease. The clinical signs associated with seropositivity for *L. infantum* (i.e., weight loss, *P* = 0.023; lymphadenomegaly*, P* = 0.002; gingivostomatitis, *P* = 0.017; and oral ulcers, *P* = 0.002) agree with the FeL clinical presentation described previously [11]. Conversely, dermatological lesions, although defined as common FeL clinical signs [17, 74], were not predominant among seropositive cats examined in this study. Thus, FeL should be considered as a polysymptomatic disease that may or not present dermatological signs. Furthermore, the laboratory abnormalities statistically related to seropositive animals (i.e., hypoalbuminemia, *P* = 0.002; reduced A/G ratio,* P* < 0.0001; increased total globulins, *P* < 0.0001; increased total proteins,* P* = 0.004; leukocytosis,* P* = 0.028; thrombocytosis, *P* = 0.006) are consistent with the pathogenesis of infection and reflect the pathological findings typically observed in CanL [18]. These outcomes are supported by previous data [11] and underscore the importance of investigating hypergammaglobulinemia as a possible laboratory alteration indicative of FeL. Nevertheless, clinical signs and hematological abnormalities reported here should be interpreted prudently considering the possible occurrence of concomitant diseases and/or co-infections within the studied feline population.

## Conclusions

This study provides, for the first time, a large-scale epidemiological survey on FeL, highlighting the circulation of *L. infantum* among domestic cats, especially shelter/free-roaming and FIV-infected animals, living in CanL endemic countries of the Mediterranean Basin. Some clinical signs and clinicopathological abnormalities were statistically related to *Leishmania* infection in the studied cat populations. Therefore, FeL should be included in differential diagnoses for feline patients with suggestive clinical signs and clinicopathological abnormalities when they live in or have traveled to regions where the disease is endemic. Under the above circumstances, the data substantiate the need for preventive measures using proper repellents for cats during the sand fly transmission season.

## Data Availability

All data supporting the main conclusions of this study are included in the manuscript. Raw data are available from the corresponding author upon reasonable request. No datasets were generated or analyzed during the current study.

## Abbreviations

DNA: Deoxyribonucleic acid
PCR: Polymerase chain reaction
qPCR: Quantitative (real-time) polymerase chain reaction
CI: Confidence interval
IFAT: Immunofluorescence antibody test
ELISA: Enzyme-linked immunosorbent assay
FIV: Feline immunodeficiency virus
FeLV: Feline leukemia virus
CanL: Canine leishmaniosis
FeL: Feline leishmaniosis
kDNA: Kinetoplast deoxyribonucleic acid
IgG: Immunoglobulin G
RBC: Red blood cells
WBC: White blood cells
Hct: Hematocrit
Hgb: Hemoglobin
PLT: Platelet count
A/G ratio: Albumin/globulin ratio
